# The sandwich technique used for correction of pectus carinatum combined with Harrison sulcus

**DOI:** 10.1038/s41598-024-66308-2

**Published:** 2024-07-05

**Authors:** Ziyin Shang, Xianlun Duan, Chun Hong, Yuan Si

**Affiliations:** 1 Department of Pediatric Thoracic Surgery, Guangdong Women and Children Hospital, Guangzhou, 511442 Guangdong Province China; 2 Department of Thoracic Surgery, Anhui Province Children’s Hospital, Hefei, 230022 Anhui Province China; 3 Department of Neurology, The Third Affiliated Hospital of Sun Yat-Sen University, Guangzhou, 510630 Guangdong Province China

**Keywords:** Pectus carinatum, Harrison sulcus, Paediatrics, Diseases, Medical research, Paediatric research

## Abstract

We aimed to investigate the feasibility of the sandwich technique to treat pectus carinatum combined with Harrison sulcus. We retrospectively analysed the clinical data of 38 paediatric patients with pectus carinatum combined with Harrison sulcus treated from June 2015 to October 2022. All the patients underwent surgery using the sandwich technique. The surgical conditions and postoperative outcomes of the patients and the satisfaction score of family members were analysed. Overall, the patients had a mean duration of surgery of 179.05 ± 36.01 min, intraoperative blood loss of 10.03 ± 2.77 mL, postoperative hospital stay of 6.89 ± 0.73 days, and postoperative satisfaction score of 89.4 ± 4.6. The incidence of surgical complications was 7.89%. The internal fixation stents were removed in 22 patients, and there was no recurrence during a follow-up 371.4 ± 6.3 days post-stent removal. These results were satisfactory. The use of the sandwich technique to treat this condition does not reduce the volume of the thorax after the procedure and results in an aesthetically pleasing incision, less complications, and fast postoperative recovery. Thus, it is a safe and effective method that is worthy of being promoted for clinical application.

## Introduction

Pectus carinatum is a common paediatric chest wall deformity that affects the growth and development, cosmetic appearance, and psychological health of patients. The prevalence of pectus carinatum in the general population is 0.3–0.7%, and it is the second most common chest wall deformity in children after pectus excavatum^1,2^. The deformity tends to progressively worsen as paediatric patients grow older; thus, it is of great importance to actively correct pectus carinatum. At present, the most commonly used treatment methods are orthopaedic braces^3,4^ and minimally invasive surgical treatment^5,6^. An increasing number of paediatric patients with pectus carinatum and their parents choose to be treated with orthopaedic braces due to the advantages such as non-invasiveness, no surgical scar, and low treatment cost; moreover, good outcomes have been achieved^6,7^. However, for some complex chest wall deformities, such as pectus carinatum combined with Harrison sulcus, conservative treatment is often ineffective. Harrison sulcus is a localised indentation at the anterior lower chest wall roughly along the 6th rib and above the costal arch, and it is sometimes combined with rib flare; it is usually bilateral but can also occur unilaterally. It can be associated with other congenital chest deformities such as pectus carinatum and pectus excavatum. In an upright position, the groove is observed to start from the xiphoid and becomes shallower, eventually disappearing as it approaches the midaxillary line^8^. In this study, we investigated the effectiveness of a minimally invasive sternal compression procedure combined with the Nuss procedure in treating paediatric patients with pectus carinatum combined with Harrison sulcus. This procedure is also known as the sandwich technoique. The relevant factors, such as the feasibility of the surgical techniques, precautions, and postoperative effectiveness, are summarised for clinical reference.

## Data and methods

### General data

This study was approved by the Ethical Review Committee of Guangdong Women and Children Hospital (No. 202301142), which waived the requirement for informed consent owing to the retrospective nature of the study. All the study protocols were performed in accordance with the principles of the Declaration of Helsinki.

**Figure 1:**
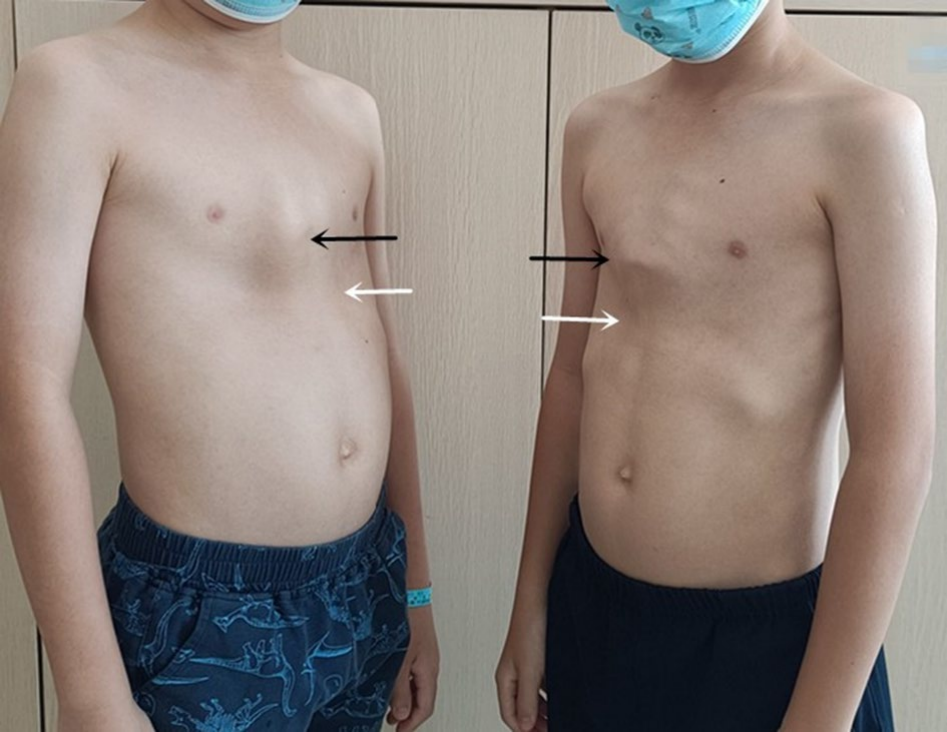
Preoperative external appearance of the chest wall of a paediatric patient (black arrows: protruding pectus carinatum, white arrows: Harrison sulcus).

**Figure 2:**
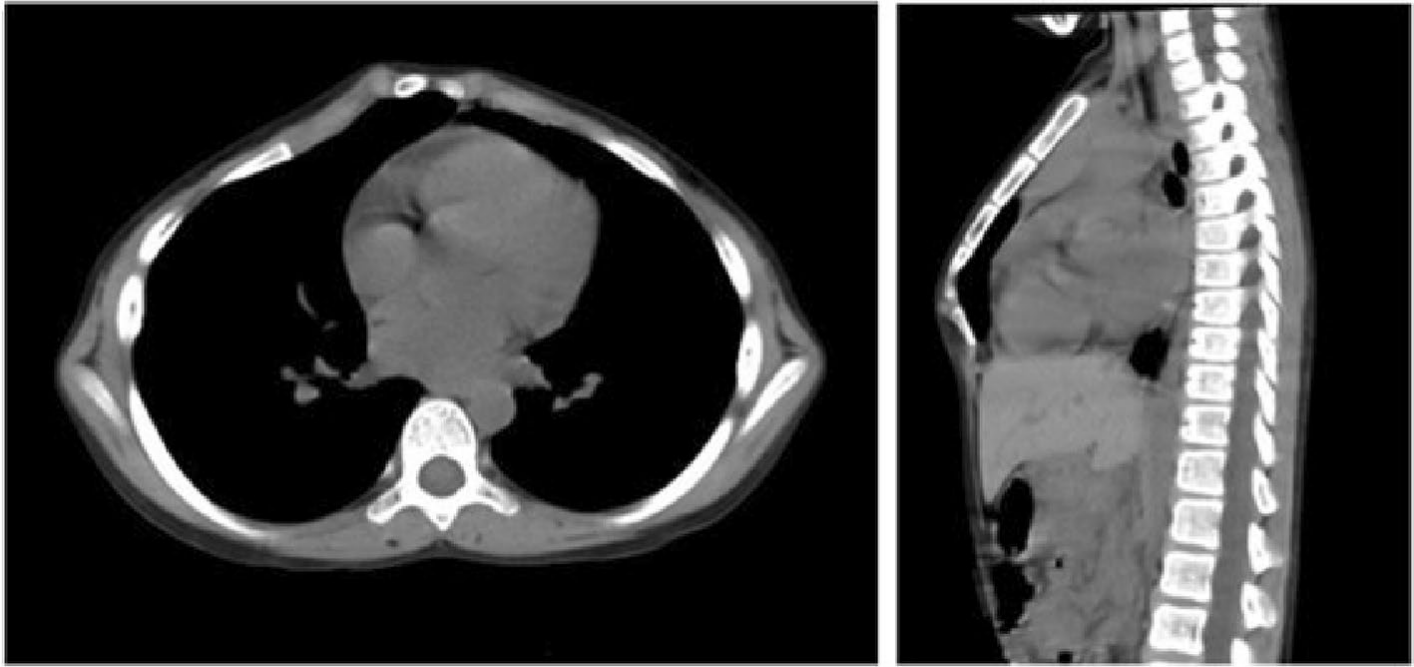
Preoperative CT scan of the chest of the paediatric patient, showing the sternum protruding forward that forms an angular deformity.

Thirty-eight paediatric patients, including 36 males and two females, treated for pectus carinatum combined with Harrison sulcus from June 2015 to October 2022 were included. Patients were aged 8–18 years (average 12.89 ± 1.84 years), weighed 44.18 ± 9.91 kg, and had a Haller index of 2.13 ± 0.14. Eleven of them had previously worn orthopaedic braces for pectus carinatum or had undergone vacuum bell therapy for pectus excavatum and underwent surgery after conservative treatment had failed. All paediatric patients underwent routine electrocardiography (ECG), echocardiography, frontal and lateral chest radiography, and chest computed tomography (CT) examination before the surgery. The Haller index was measured, and the cardiopulmonary function, severity of the thoracic deformity, and presence of comorbidities were examined. The external appearance and CT examination for a typical case of thoracic deformity are shown in Figs. 1 and 2.

The inclusion criteria were as follows: (a) age of 8–18 years; (b) pure pectus carinatum with obvious Harrison sulcus when pressing the highest point of the raised sternum back to the normal position; c) patients who underwent a minimally invasive sternal compression procedure combined with the Nuss procedure and completed regular follow-ups at 1, 4, 12, and 24 months after the surgery; and d) complete clinical and postoperative follow-up data.

The exclusion criteria were as follows: (a) age less than 8 or more than 18 years; (b) pectus carinatum caused by cardiopulmonary diseases or mediastinal tumour; (c) pectus carinatum after congenital heart disease-related or other chest wall surgery; or (d) patients cured by conservative treatment (orthopaedic braces or vacuum bell for pectus excavatum).

### Surgical methods

#### Indications for surgery

Patients were indicated for surgery when two or more of the following criteria were met: (a) pulmonary function, ECG, and echocardiogram indicated abnormalities such as restrictive, obstructive airway disease; (b) progression of deformity or combination with obvious symptoms; (c) failure of conservative treatment (orthopaedic braces or vacuum bell for pectus excavatum); and (d) deformity of external appearance intolerable to the patients or their parents.

#### Surgical operation

An appropriate type of orthopaedic plate was selected. The length of the pressing steel plate was centred on the highest point of the sternal prominence. The lengths of both the mid-axillary line and the anterior axillary line were measured using a skin ruler, and the steel plate's length should fall within this range. The final length of the steel plate is determined based on the rib position where the steel plate will be secured.

The thoracic cage was pressed at the highest protruding point of the sternum until the desired height was achieved, and the plate was bent into an arc corresponding to the external appearance of the thoracic cage at this point. A transverse incision of about 2 cm was made in the mid-axillary line, followed by incision of the skin and subcutaneous fat layer by layer and separation of the muscular layer to expose the ribs. The fixing pieces for the plate were fixed on the corresponding ribs with stainless-steel wires without tightening the wires. Careful dissection was performed to avoid damaging the pleura and accidentally entering the thoracic cavity. A tunnel was made under the muscular layer towards the highest protruding point of the thoracic cage. An introducer was placed through the tunnel, and a silicone tube was introduced for plate traction. The shaped plate was brought through the subcutaneous tunnel to the predetermined position by the silicone tube.

The upward plate was bent using the same method. An introducer was placed under direct thoracoscopic view, followed by blunt dissection of the tissues behind the sternum, passing the introducer through the lowest point of the Harrison sulcus to the contralateral incision and then pulling in the pre-bent plate.

The downward plate was pressed down to reach the desired height, and the two ends of the plate were tied to the fixing pieces with stainless steel wires. Finally, the steel wires at both ends of the fixing plate were tightened to secure them on the adjacent costal cartilage of the ribs. The minimally invasive sternal compression procedure was then completed.

**Figure 3:**
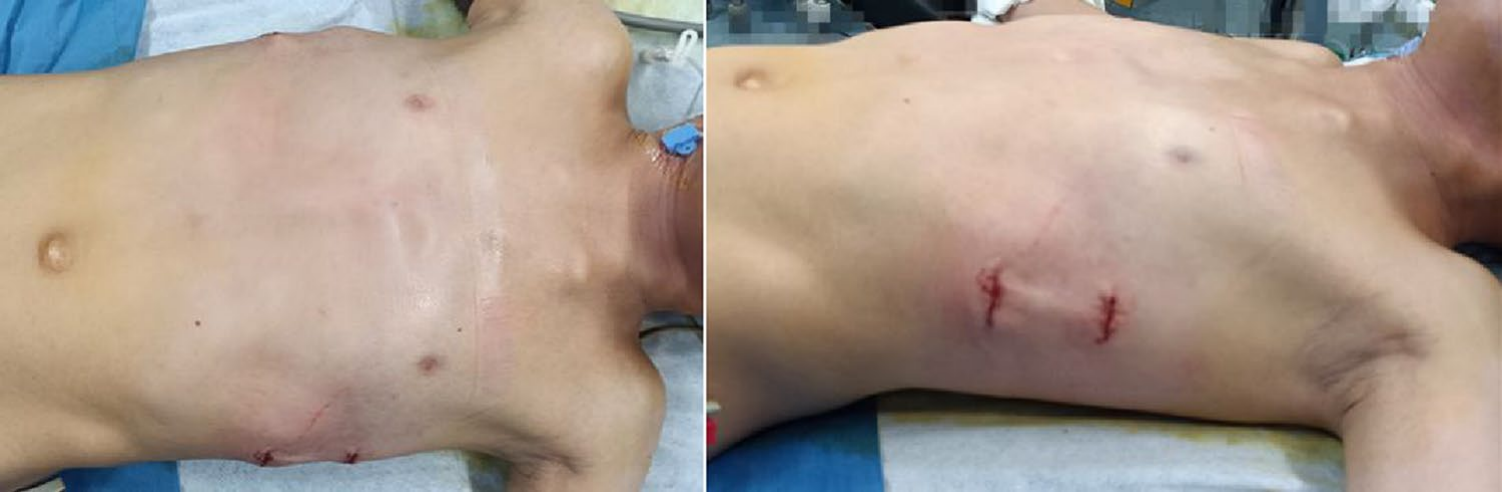
Postoperative external appearance after a minimally invasive sternal compression procedure combined with the Nuss procedure, showing a flat chest wall with good aesthetics.

**Figure 4:**
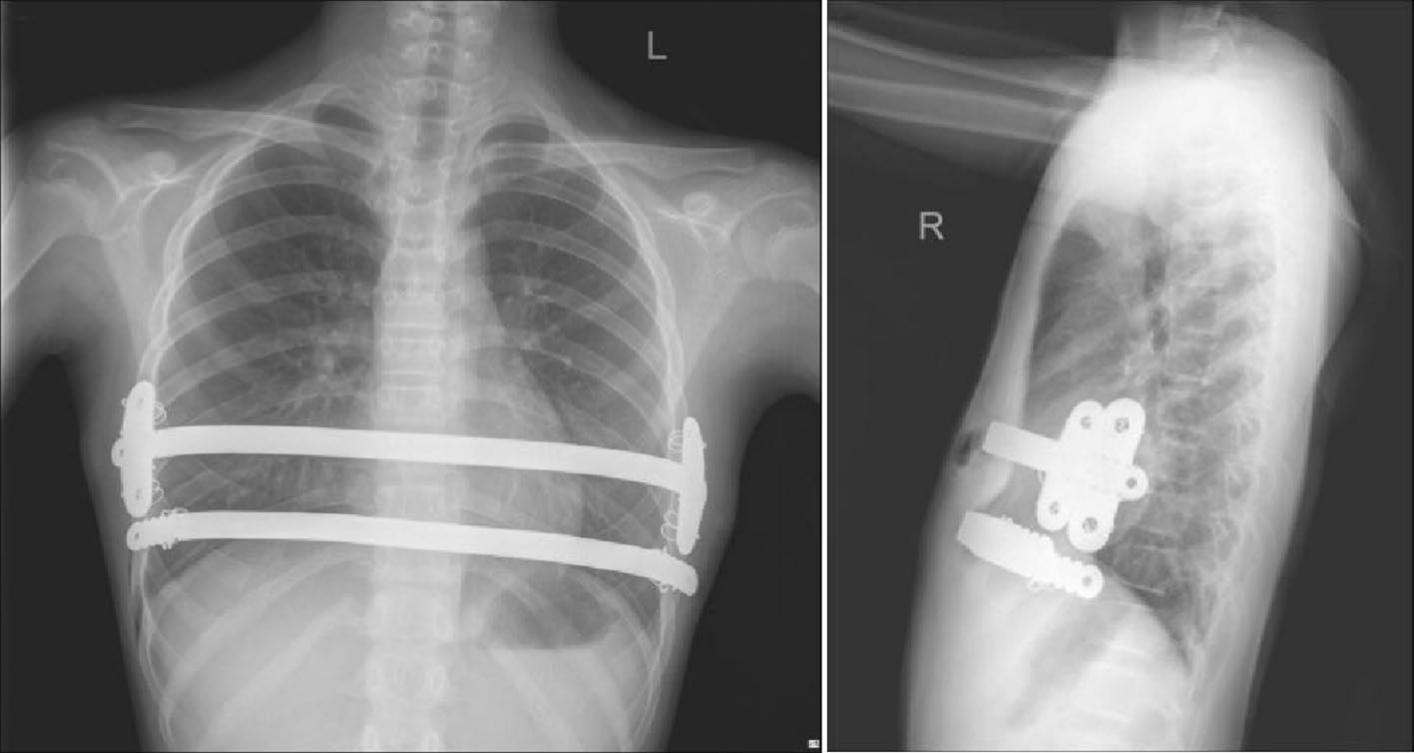
Postoperative frontal and lateral radiographic films of the chest, showing that the sternum is flat and straight, and the plate and fixing pieces are well-positioned.

The upward plate was then flipped so that its curved arch was facing upward to push the indented area of the Harrison sulcus upwards, which straightened the anterior chest wall indentation. The postoperative thoracic external appearance and radiographic films of a paediatric patient are shown in Figs. 3 and 4.

### Observation of indicators

Perioperative indicators such as surgery duration, intraoperative blood loss, and occurrence of postoperative complications were recorded. In addition, satisfaction regarding the orthopaedic correction outcome was rated with reference to the following five criteria before each paediatric patient was discharged from the hospital: (1) straightening of the sternal line indicated by the frontal and lateral chest radiographs during postoperative review; (2) satisfaction with changes in the external appearance of the thoracic cage after the surgery; (3) satisfaction of the patients or their parents and physicians with postoperative orthopaedic correction outcomes; (4) presence of postoperative complications and physical appearance after healing; and (5) satisfaction with the perioperative anaesthesia and medical care. Twenty points were allocated for each of the five criteria, and the total score was out of 100 points. The physician and the parents of each paediatric patient rated these items separately, and the average of the two values was taken as the final satisfaction score.

## Results

**Figure 5:**
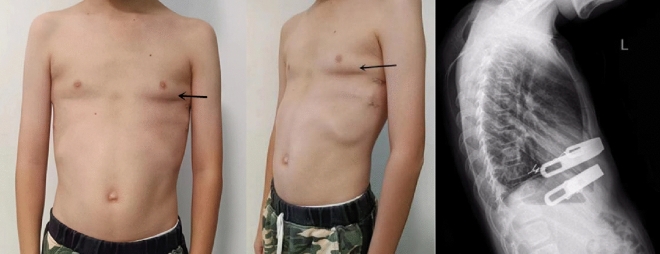
This paediatric patient was treated with a plate tied by stainless-steel wires without using fixing pieces. The plate is slightly displaced 3 months after the surgery. The arrow marks the bulging plate.

**Figure 6:**
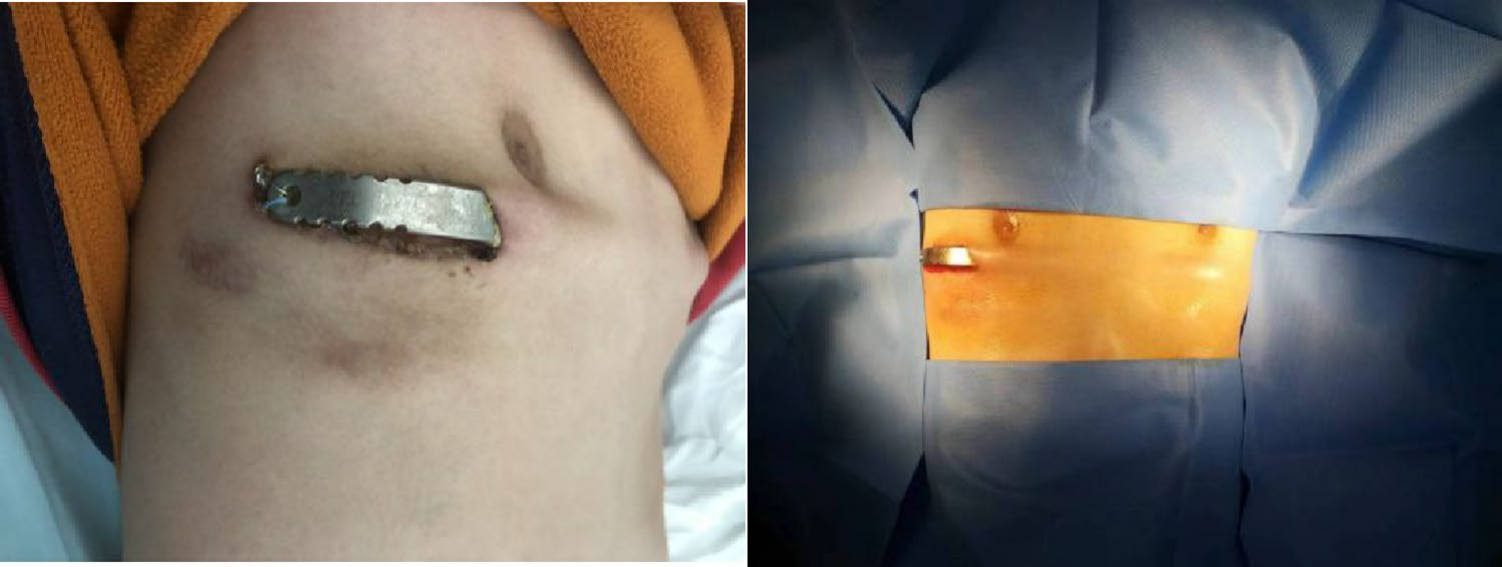
Incision dehiscence and plate exposure.

All paediatric patients successfully completed the procedure, with a surgical duration of 179.05 ± 36.01 min, intraoperative blood loss of 10.03 ± 2.77 mL, and an average postoperative hospital stay of 6.89 ± 0.73 days. To effectively relieve pain, 30 patients (78.9%) received ropivacaine intraoperative regional block analgesia. All patients (100%) received sufentanil plus nonsteroidal anti-inflammatory drugs by patient-controlled-analgesia after surgery; twenty-five patients (65.8%) were administered intravenous or oral analgesics after surgery. In two cases with plates fixed with stainless-steel wires on both sides instead of fixing pieces, the stainless-steel wires loosened after the surgery and caused slight displacement of the plates. However, the external appearance of the sternum was satisfactory without protruding, and thus no treatment was administered (Fig. 5). In one case with serious incision dehiscence and exposure of one end of the compression plate, the drug treatment was ineffective, and the compression plate was removed 11 months after the surgery. Fortunately, the orthopaedic correction of the protruding chest wall was satisfactory (Fig. 6). All patients were followed up regularly at 1, 4, 12, and 24 months after discharge. Plates were removed in 22 patients who were followed up for 1 year after removal. There were no patients with recurrence, and the satisfaction score was 89.4 ± 4.6 for the treatment outcomes. Detailed clinical data are shown in Table 1.

**Table 1:** Clinical data of 38 paediatric patients with pectus carinatum combined with Harrison sulcus.

Clinical data	Values
Sex (male/female)	36/2
Age (years)	8–18 (12.89 ± 1.84)
Body weight (kg)	23.0–68.5 (44.18 ± 9.91)
Haller index	1.87–2.27 (2.13 ± 0.14)
Duration of surgery (min)	107–280 (179.05 ± 36.01)
Intraoperative blood loss (mL)	5–20 (10.03 ± 2.77)
Postoperative hospital stay (d)	6–9 (6.89 ± 0.73)
Complications [n(%)]	3 (7.5)
Incision dehiscence, plate exposure	1 (2.5)
Loosening of implants	2 (5.0)
Satisfaction score	77.5–99.0 (89.4 ± 4.6)

## Discussion

Orthopaedic braces for pectus carinatum and vacuum bell therapy for pectus excavatum are conservative therapies for chest wall deformities and have become the preferred treatment options for patients with pectus excavatum and pectus carinatum owing to their advantages, including non-invasiveness, no surgical scarring, and low treatment cost^9^. For most patients with pectus carinatum combined with Harrison sulcus, favourable outcomes can be achieved using orthopaedic braces combined with vacuum bell therapy, but for other patients these treatments are ineffective. Eleven paediatric patients in this group, who experienced unsatisfactory outcomes following conservative treatment, underwent surgery. The reasons for the failure of conservative treatment were as follows: (a) poor compliance of the parents with medical advice regarding the use of the orthopaedic braces and vacuum bell therapy, and insufficient wearing time or irregular wearing due to patient aversion and resistance to the treatment, which ultimately led to treatment failure; (b) poor thoracic compliance and stiffness and poor elasticity of the bones resulting in failure of conservative treatment in older paediatric patients or adults^6^. Both the minimally invasive sternal compression procedure and the Nuss procedure are established procedures for the treatment of pectus carinatum and pectus excavatum that have been validated worldwide^10,11^. When a minimally invasive sternal compression procedure is used to treat pectus carinatum, additional indentation may occur around the site of sternal compression^12^. For pectus carinatum combined with Harrison sulcus, the Harrison sulcus is exacerbated when the sternum is compressed during treatment. This not only results in failure to improve external appearance but also a reduction in thoracic volume. The sandwich technique is an effective surgical method with plate supports under the sternum, which to some extent avoids excessive compression of the anterior chest wall with the plate above the sternum leading to pectus excavatum. At the same time, this surgical approach has the advantages of less trauma, less intraoperative blood loss, fast postoperative recovery, and pleasing postoperative external appearance of the chest wall.

The length of the steel plate, the distance between the two plates, the selection of the entrance and exit points, and the location of the incisions need to be carefully designed. The two plates should not be too close to each other to allow adequate space for the fixing pieces. In two cases in this group, the plates were placed too close to each other. Hence, the fixing pieces could not be placed, and thus the plates were tied directly with stainless-steel wires, which resulted in the loosening of the wires and plate displacement after surgery. The fixing pieces were also not placed in another case with incision dehiscence and plate exposure. The causes of complications were analysed and were as follows: (1) the plate was placed under the fat layer, and the patient was slim with a thin subcutaneous fat layer and a lack of a chest muscular layer to protect the plate; (2) the patient was in adolescence with rapid growth in height and the thoracic cage, which caused the plate to exert more tension on the skin from the inside out, eventually leading to skin abrasion by the plate and plate exposure. Based on the lessons learned, we placed the plates under the muscular layer in the subsequent surgeries. The placement of two plates often causes high skin tension, and the plates are prone to exposure once infection or rejection occurs, which leads to the risk of premature removal of the plates. The thicker muscular and fat layers wrap the plates and fixing pieces tightly, and it is easier to heal after regular dressing changes in case of infection. As the intersection of the lowest point of indentation and the sternum is often located at the xiphoid process, the separation of the tissues behind the sternum by the introducer needs to be performed under direct thoracoscopic view to avoid damage to the diaphragm.

Afterundergoing surgery using the sandwich technique, paediatric patients often feel severe pain that can cause postoperative complications such as insomnia, fear of excreting sputum by coughing due to the fear of pain, which leads to postoperative pneumonia and atelectasis, and scoliosis due to the long-term protective posture of paediatric patients. Therefore, effective surgical analgesia is extremely important for the postoperative recovery of paediatric patients. All cases in our group received multimodal analgesia^13^, where ropivacaine was used for a regional block during anaesthesia, sufentanil combined with non-steroidal anti-inflammatory drugs was administered intravenously for 2–3 days after the surgery for analgesia, and additional intravenous or oral analgesics, such as acetaminophen, ibuprofen, and diclofenac, were administered in cases of severe pain.

Although the sandwich technique is minimally invasive and effective, the age and indications for surgery need to be strictly controlled. We limited the age of enrolment as 8–18 years. For prepubertal paediatric patients with pectus carinatum, their bones are still soft, the thoraxes are easily shaped, and the treatment outcomes are satisfactory with orthopaedic braces and vacuum bell therapy. Undergoing surgery before puberty is associated with a risk of recurrence and can affect the growth of the ribs of paediatric patients. However, in adulthood, the patients have stiff bones and poor thoracic elasticity, and more support is required to lift the thoraxes forward, which can easily lead to unsatisfactory orthopaedic correction outcomes. Therefore, we believe that early adolescence is the best time to perform this surgery. One case in this group was operated on at the age of 8 years due to the strong desire of the patient and parents for surgery, and the rest of the paediatric patients were operated on at the age of 10 years or above. For some congenital cardiopulmonary diseases, such as congenital diaphragmatic hernia, retrosternal hernia, and lobar emphysema, secondary pectus carinatum formed by forward extrusion of the sternum can be initially treated with braces after treating the primary disease.

## Conclusion

In conclusion, pectus carinatum combined with Harrison sulcus is a complex chest wall deformity, particularly in older adolescents, and the outcomes of conservative treatment are often unsatisfactory. The use of the sandwich technique is a safe and effective treatment option for paediatric patients with this condition and is worthy of being promoted for clinical application. However, the age of surgical patients and indications for surgery should be strictly controlled.

## Data Availability

Data that support the findings of this study are available upon reasonable reques from one of the study authors (Ziyin Shang).

## References

[1] Musters GD, Oomen MWN, Zwaveling S, de Jong JR, de Beer SA (2019). Dynamic compression brace for pectus carinatum: 5 years on. Ned. Tijdschr. Geneeskd..

[2] Brochhausen C (2012). Pectus excavatum: History, hypotheses and treatment options. Interact. Cardiovasc. Thorac. Surg..

[3] Shang ZY (2021). Experience of orthopedic braces for pectus carinatum in children. J. Thorac. Cardiovasc. Surg..

[4] Port E, Hebal F, Hunter CJ, Malas B, Reynolds M (2018). Measuring the impact of brace intervention on pediatric pectus carinatum using white light scanning. J. Pediatr. Surg..

[5] Özkaya M, Bilgin M (2018). Minimally invasive repair of pectus carinatum: A retrospective analysis based on a single surgeon’s 10 years of experience. Gen. Thorac. Cardiovasc. Surg..

[6] Shang Z, Hong C, Duan X, Li X, Si Y (2021). Orthotic Bracing or Minimally Invasive Surgery? A summary of 767 pectus carinatum cases for 9 years. BioMed Res. Int..

[7] van Braak H, de Beer SA, Zwaveling S, Oomen MWN, de Jong JR (2024). Ravitch surgery or dynamic compression bracing for pectus carinatum: A retrospective cohort study. Ann. Thorac. Surg..

[8] Yoham, A. L. & Sajjad, H. Anatomy, abdomen and pelvis: Harrison groove in *Stat*Pearls [Internet] (StatPearls Publishing, Treasure Island FL, 2022) (PMID, Jan, 2022) (2023). 32965879

[9] Obermeyer RJ (2018). Nonoperative management of pectus excavatum with vacuum bell therapy: A single center study. J. Pediatr. Surg..

[10] Abramson H (2005). A minimally invasive technique to repair pectus carinatum [Preliminary report]. Arch. Bronconeumol..

[11] Torre M (2021). Complications and trends in minimally invasive repair of pectus excavatum: A large volume, single institution experience. J. Pediatr. Surg..

[12] Park HJ, Kim KS (2016). The sandwich technique for repair of pectus carinatum and excavatum/carinatum complex. Ann. Cardiothorac. Surg..

[13] Ben-David B, Swanson J, Nelson JB, Chelly JE (2007). Multimodal analgesia for radical prostatectomy provides better analgesia and shortens hospital stay. J. Clin. Anesth..

